# Extracorporeal hemoadsorption in critically ill COVID-19 patients on VV ECMO: the CytoSorb therapy in COVID-19 (CTC) registry

**DOI:** 10.1186/s13054-023-04517-3

**Published:** 2023-06-19

**Authors:** J. W. Awori Hayanga, Tae Song, Lucian Durham, Lawrence Garrison, Deane Smith, Zsolt Molnar, Joerg Scheier, Efthymios N. Deliargyris, Nader Moazami

**Affiliations:** 1 Department of Cardiovascular and Thoracic Surgery, West Virginia University, 1 Medical Center Drive, Morgantown, WV 26506 USA; 2 University of Chicago Medicine, Chicago, USA; 3 Medical College of Wisconsin, Milwaukee, USA; 4 Franciscan Health Indianapolis, Indianapolis, USA; 5 CytoSorbents Europe, Berlin, Germany; 6 Semmelweis University, Budapest, Hungary; 7 CytoSorbents Corporation, Princeton, USA; 8 New York University School of Medicine, New York, USA

**Keywords:** Coronavirus—COVID-19, ECMO—Extracorporeal membrane oxygenation, ICU—Intensive care unit, ARDS—Acute respiratory distress syndrome, Mortality, Inflammation, Hemoperfusion, Hemoadsorption, CytoSorb

## Abstract

**Objectives:**

The CytoSorb therapy in COVID-19 (CTC) registry evaluated the clinical performance and treatment parameters of extracorporeal hemoadsorption integrated with veno-venous extracorporeal membrane oxygenation (VV ECMO) in critically ill COVID-19 patients with acute respiratory distress syndrome (ARDS) and respiratory failure under US FDA Emergency Use Authorization.

**Design:**

Multicenter, observational, registry (NCT04391920).

**Setting:**

Intensive care units (ICUs) in five major US academic centers between April 2020 and January 2022.

**Patients:**

A total of 100 critically ill adults with COVID-19-related ARDS requiring VV ECMO support, who were treated with extracorporeal hemoadsorption.

**Interventions:**

None.

**Measurements and main results:**

Baseline demographics, clinical characteristics, laboratory values and outcomes were recorded following individual ethics committee approval at each center. Detailed data on organ support utilization parameters and hemoadsorption treatments were also collected. Biomarker data were collected according to the standard practice at each participating site, and available values were compared before and after hemoadsorption. The primary outcome of mortality was evaluated using a time-to-event analysis. A total of 100 patients (63% male; age 44 ± 11 years) were included. Survival rates were 86% at 30 days and 74% at 90 days. Median time from ICU admission to the initiation of hemoadsorption was 87 h and was used to define two post hoc groups: ≤ 87 h (group-early start, *G*_E_) and > 87 h (group-late start, *G*_L_). After the start of hemoadsorption, patients in the *G*_E_ versus *G*_L_ had significantly shorter median duration of mechanical ventilation (7 [2–26] vs. 17 [7–37] days, *p* = 0.02), ECMO support (13 [8–24] vs. 29 [14–38] days, *p* = 0.021) and ICU stay (17 [10–40] vs 36 [19–55] days, *p* = 0.002). Survival at 90 days in *G*_E_ was 82% compared to 66% in G_L_ (*p* = 0.14). No device-related adverse events were reported.

**Conclusions:**

In critically ill patients with severe COVID-19-related ARDS treated with the combination of VV-ECMO and hemoadsorption, 90-day survival was 74% and earlier intervention was associated with shorter need for organ support and ICU stay. These results lend support to the concept of “enhanced lung rest” with the combined use of VV-ECMO plus hemoadsorption in patients with ARDS.

**Supplementary Information:**

The online version contains supplementary material available at 10.1186/s13054-023-04517-3.

## Introduction

Following its emergence in December 2019, the Coronavirus disease 2019 (COVID-19), a highly contagious viral illness caused by the severe acute respiratory syndrome coronavirus 2 (SARS-CoV-2), placed an unprecedented burden on intensive care units (ICUs) and health care systems around the globe. As of August 2022, the disease had claimed more than 6 million lives worldwide, rendering it the most consequential global health crisis since the influenza pandemic of 1918 [1].

During the COVID-19 pandemic, the US Food and Drug Administration (FDA) issued numerous Emergency Use Authorizations (EUAs) to expedite access to novel treatment approaches. In this vein, extracorporeal hemoadsorption with CytoSorb (CytoSorbents Corporation, Princeton, NJ, USA) was granted FDA EUA in April 2020 based on the ability to extract a broad range of inflammatory mediators, including cytokines, and to reduce hyperinflammation, a critical pathophysiologic component of severe COVID-19 illness [2, 3].

The rationale for hemoadsorption as an adjunctive therapy in critically ill COVID-19 patients with refractory respiratory failure on extracorporeal membrane oxygenation (ECMO) is to promote lung healing and recovery by reducing hyperinflammation while permitting the lungs to rest on ECMO [4]. This concept of “enhanced lung rest” centers around the ability of ECMO to ensure adequate oxygenation and CO_2_ removal while protecting from ventilator-induced lung injury (VILI), and the ability of hemoadsorption to address the ongoing hyperinflammation that may contribute to endothelial tight junction disruption and capillary leak syndrome which exacerbates injury to the lungs and other organs. The combination of these therapies may reduce the severity of illness, promote faster organ healing, and potentially improve survival [4].

The CytoSorb device is a 300-mL cartridge filled with porous polymer beads with an active adsorbent surface area of approximately 45,000 m^2^. The beads are capable of adsorbing hydrophobic substances up to 60 kDa in size from whole blood in a concentration-dependent manner. The hemoadsorption cartridge is designed for integration in extracorporeal circuits and is commonly incorporated pre- or post-filter during continuous renal replacement therapy (CRRT), in extracorporeal circuits used for ECMO or cardiopulmonary bypass (CPB), and also in stand-alone hemoperfusion machines [5].

The CytoSorb® Therapy for COVID-19 patients (CTC) registry was a multicenter study designed to collect high fidelity, patient-level data from critically ill COVID-19 patients with respiratory failure on VV ECMO also treated with hemoadsorption under FDA EUA. Preliminary results of the first interim analysis on 52 patients have previously been published [6]. We now report the final results of the CTC registry and include an exploratory analysis evaluating the timing of hemoadsorption therapy and its influence on outcomes.

## Methods

### Patient population and treatment parameters according to FDA Emergency Use Authorization

The study cohort comprised 100 critically ill COVID-19 patients treated using a combination of VV ECMO and CytoSorb at 5 US centers. As we have previously published [6], the decision to use hemoadsorption therapy was at the discretion of the treating physicians abiding by the criteria of the EUA Instructions For Use [7]. The EUA did not specify laboratory criteria for a hyperinflammatory state and treating physicians relied on either inflammatory parameters such as ferritin (> 1000 ng/mL), C-reactive protein (CRP; > 50 mg/L), and/or elevated cytokines such as interleukin-6 (IL-6 > 500 pg/mL) or supportive clinical evidence to initiate treatment. Prior to clinical use, all institutions were provided with on-site education and training on the device by the manufacturer (CytoSorbents Corporation, USA). The hemoadsorption device was integrated into a shunt circuit (i.e., post-pump, through the bottom and up through the top of the device, and back pre-pump) in parallel to the main ECMO circuit. Flow rates ranged from 400 to 600 mL per minute. Heparin was used for anticoagulation in all cases [6]. Per the EUA instructions, patients received 72 h of continuous hemoadsorption treatment. The adsorbers were changed every 12 h on the first day followed by a device exchange every 24 h thereafter. Treatment decisions were guided by simultaneous evaluation of the patients’ clinical condition in order to determine whether therapy should be continued beyond 72 h. Concomitant medications that could potentially be extracted by the device, such as remdesivir or steroids, were administered 1–2 h before restarting hemoadsorption to allow drug distribution into tissues.

### CTC registry design

The CTC registry was a multicenter, observational study enrolling critically ill COVID-19 patients who received hemoadsorption therapy under FDA EUA (ClinicalTrials.gov Identifier: NCT04391920). All participating centers obtained approval from their respective Institutional Review Boards for retrospective de-identified patient data collection from medical records and entry into the CTC registry electronic database, thereby waiving the need to obtain individual patient consent (Additional file 1: Table S1). Data collected included demographics, medical history, COVID-19-related medications and adverse events. Baseline (i.e., before the start of CytoSorb therapy) inflammatory biomarkers (i.e., IL-6, ferritin, CRP), serum D-dimer levels and ventilation specific parameters (PaO_2_/FiO_2_, PaCO_2_, Positive End Expiratory Pressure; PEEP, peak inspiratory pressure; Ppeak and driving pressure) were recorded. Utilization parameters for hemoadsorption and other organ support therapies were also recorded. All procedures were followed in accordance with the ethical standards of the responsible committee on human experimentation (institutional or regional) and with the Helsinki Declaration of 1975.

### Measurements and patient outcomes

Patient follow-up was extended until death or hospital discharge. Mortality was the primary outcome. Consistent with the approach of the Extracorporeal Life Support Organization (ELSO) registry that reports mortality at 90 days, we performed a comparable time-to-event analysis to evaluate mortality over time to 90 days following ICU admission. Secondary outcomes were the duration of mechanical ventilation (MV), ECMO, vasopressor support, ICU stay and the need for continuous renal replacement therapy (CRRT) after the start of CytoSorb therapy. Additionally, we performed a post hoc exploratory analysis by using the median time from ICU admission to initiation of hemoadsorption therapy to dichotomize the study population into two equally sized groups (*n* = 50 each): ≤ 87 h, group-early start (*G*_E_); and > 87 h, group-late start (*G*_L_). Ninety day mortality and secondary outcomes were also evaluated in a similar fashion as in the entire cohort (i.e., measured after the start of hemoadsorption therapy) in both subgroups.

### Statistical analysis

Data were summarized with counts and percentages for categorical data; means, standard deviations, medians, interquartile ranges, minimums and maximums for continuous data, and Kaplan–Meier estimates for time-to-event data. For comparisons between the early versus late start groups, Chi-square test or Fisher's exact test was used for categorical variables, as appropriate. *T*-tests or Wilcoxon rank sum tests were used for continuous variables, as appropriate. We used log-rank tests to compare time-to-event variables. No adjustments for multiple comparisons were planned for these exploratory analyses. A *p*-value < 0.05 was used as the threshold for statistical significance. All statistical analyses were performed using SAS v9.4 (SAS, Cary, NC, USA).

## Results

A total of 100 critically ill patients meeting the EUA clinical criteria of life-threatening respiratory failure treated with combined ECMO and hemoadsorption therapy at 5 US centers between April 2020 and January 2022 comprised the study cohort.

### Demographics and baseline physiological parameters

Demographic data and baseline characteristics for the entire study group as well as for early and late treatment subgroups are depicted in Table 1. Patients had a mean age of 44 years and 63% were male. All patients had evidence of hyperinflammation before the start of hemoadsorption indicated by high ferritin (median 1132 [618–2592] ng/mL) and/or CRP (median 116 [43–234] mg/L) levels. Most patients had moderately elevated D-dimer levels (median 2.11 [1.29–5.57] ug/mL. Patients had severe respiratory failure as indicated by the need for VV ECMO, and baseline median values of PaO_2_/FiO_2_ ratio of 73 [62–154] mmHg, PEEP of 14 [10–15] cm H_2_O, and *P*_peak_ of 29 [25–35] cm H_2_O.

**Table 1:** Demographics and baseline characteristics

	Total *n* = 100	*G*_E_ *n *= 50	*G*_L_ *n* = 50	*p-*value
Age (Years)	44 ± 11	41 ± 10	47 ± 10	0.004
Male/Female	63/37	29/21	34/16	0.300
SOFA	6 ± 4	5.93 ± 4.65	6.10 ± 3.56	0.807
BMI (kg/m^2^)	34.4 ± 6.3	35.4 ± 6.0	33.4 ± 6.5	0.121
Hypertension, *n* (%)	38 (38)	21 (42)	17 (34)	0.410
Diabetes, *n* (%)	25 (25)	10 (20)	15 (30)	0.248
Other comorbidities, *n* (%)	19 (19)	24 (48)	14 (28)	0.039
Lactate (mmol/L)	2.2 [1.2–2.5]	2.5 [0.9–3.1]	2.0 [1.2–2.38]	0.401
PaO_2_/FiO_2_ (mmHg)	73 [62–154]	73 [59–128]	81 [62–170]	0.595
PaCO_2_ (mmHg)	52 [46–62]	48 [41–54]	56 [46–67]	0.115
PEEP (cm H_2_O)	14 [10–15]	16 [14–16]	12 [10–14]	0.027
Ppeak (cm H_2_O)	29 [25–35]	37 [29–39]	28 [25–31]	0.011
Driving pressure (cm H_2_O)	14 [10–18]	23 [21–25]	12 [9–18]	0.030
D-Dimer (mcg/mL)	2.11 [1.29–5.57] (*n* = 26)	2.01 [1.51–3.97] (*n* = 12)	4.51 [1.25–78] (*n* = 14)	0.368
CRP (mg/dL)	11.60 [4.25–23.40] (*n* = 32)	13.1 [9.5–20.3] (*n* = 9)	8.7 [3.2–26.9] (*n* = 23)	0.4892
Ferritin (ng/mL)	1132 [618–2592] (*n* = 26)	1339 [631–1927] (*n* = 10)	1061 [405–2768] (*n* = 16)	0.712
MV before ECMO (h)	19.5 [7–43]	16 [5–25]	39 [12–87]	0.001
MV before HA (h)	25 [13–79]	16 [5–29]	79 [21–146]	< 0.001
ECMO before HA (h)	0 [0–5]	0 [0–1]	1 [0–78]	< 0.001
ICU admission to ECMO (h)	58 [35–138]	37 [17–41]	138 [87–231]	< 0.001
ICU admission to HA (h)	86 [38–186]	38 [19–42]	186 [119–304]	< 0.001

Data are presented as *n* (%), median [interquartile range] or mean ± standard deviation as appropriate

Patients in the *G*_L_ (late initiation of CytoSorb > 87 h from ICU admission) group were older and had fewer comorbidities compared with the *G*_E_ (early initiation of CytoSorb ≤ 87 h from ICU admission) group. In *G*_E_ hemoadsorption was started almost immediately following cannulation and the initiation of VV ECMO, while there was a significant delay in *G*_L_ (Table 1). There were no other significant differences in demographics or baseline biomarker levels. Patients in *G*_E_ required significantly higher PEEP, *P*_peak_, and driving pressure at baseline (Table 1). The PaO_2_/FiO_2_, PaCO_2_ and lactate levels did not differ significantly between the groups.

Approximately 90% of patients received at least one medication for treatment of COVID-19 (i.e., anakinra, azithromycin, betamethasone, convalescent plasma, dexamethasone, hydrocortisone, hydroxychloroquine, methylprednisolone, prednisolone, remestemcel-l, ritonavir, remdesivir, lopinavir, sarilumab, siltuximab and tocilizumab) before being placed on ECMO, with 45% of patients continuing to receive treatment during ECMO.

### Timing of initiation of organ support therapies

Overall, VV ECMO and CytoSorb were initiated relatively early in patients that failed mechanical ventilation (MV) and standard of care measures such as proning and corticosteroids. Median duration of MV to initiation of ECMO was 19.5 [7–43] h, with generally concurrent initiation of CytoSorb: 0 [0–5] h from ECMO start (Table 1). However, compared to G_L_ patients, those in the G_E_ group started ECMO and CytoSorb significantly earlier following both ICU admission and the initiation of MV. The time interval between ECMO start and initiation of hemoadsorption treatment specifically was also shorter in the G_E_ group (Table 1).

### Need and duration of organ support therapies

**Fig. 1:**
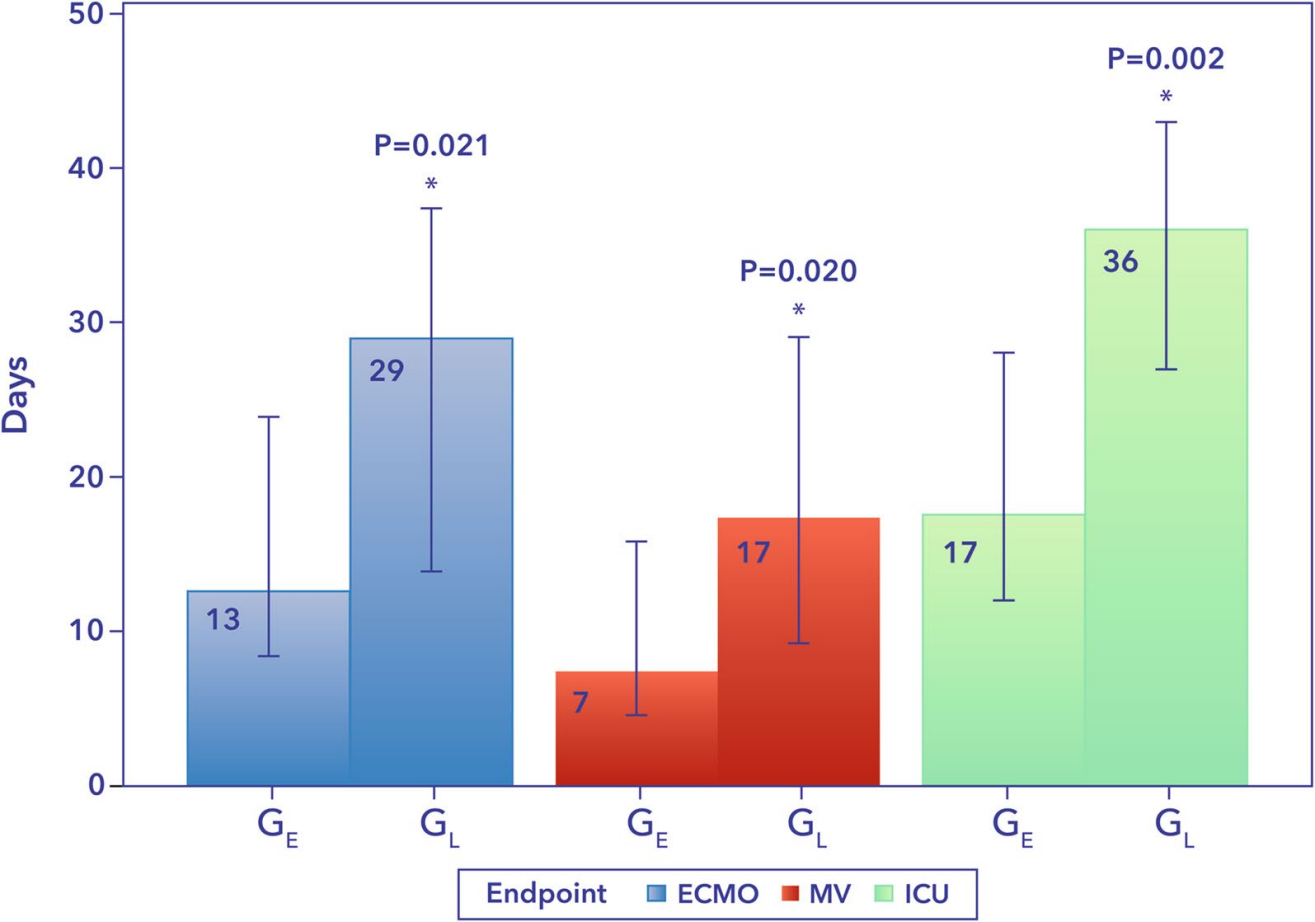
Days on organ support and ICU stay in *G*_E_ and *G*_L_. *G*_E_, early group; *G*_L_, late group; ECMO, extracorporeal membrane oxygenation; MV, mechanical ventilation; ICU, intensive care unit

Overall, earlier initiation of VV ECMO and CytoSorb was associated with significant reductions in the duration of organ support. After the start of hemoadsorption, patients in G_E_ required significantly shorter ECMO support than those in G_L_ (median 13 [7–31] vs 29 [10–47] days, respectively, *p* = 0.02) and had needed shorter duration of MV (median 7 [2–26] vs. 17 [7–37] days, *p* = 0.02) (Fig. 1). ICU stay evaluated after the start of hemoadsorption was also significantly shorter in G_E_ compared with G_L_ (17 [10–40] vs. 36 [19–55] days, respectively, *p* = 0.002) (Fig. 1).

Despite the same number of patients requiring vasopressors in both groups (76%), the G_L_ group tended to require vasopressor support for longer and also had higher final cumulative fluid balance (Table 2). Need for CRRT (30% in *G*_E_ vs. 22% in *G*_L,_ *p* = 0.36), and duration: *G*_E_ (30 [7–53] vs. 5 [4–24] days, *p* = 0.12, showed no significant differences between the groups.

**Table 2:** Clinical course parameters

	Total *n* = 100	*G*_E_ *n* = 50	G_L_ *N* = 50	*p-*value
Vasopressor support, *n* (%)	76 (76)	38 (76)	38 (76)	1.000
Vasopressor support after HA start (days)	5 [3–21]	4 [1–17]	7.5 [4–21]	0.128
Cumulative fluid balance (mL)	1988 [− 933 to 4727]	990 [− 993 to 3894]	2322 [− 969 to 5957]	0.336

Data are presented as *n*(%) and median[interquartile range]

### Vital status

**Fig. 2:**
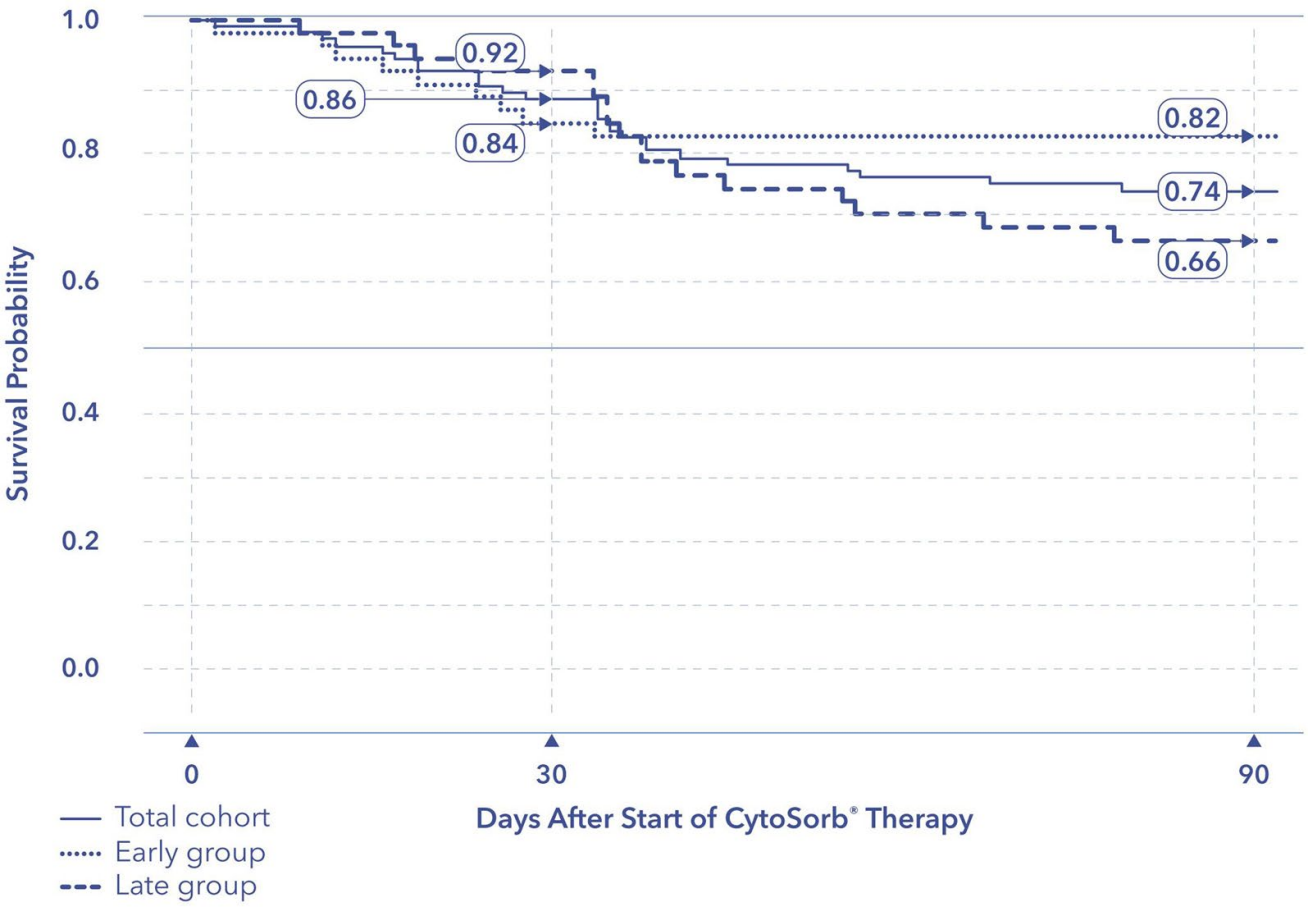
Kaplan–Meier plot for probability of survival from admission to ICU to 90 days in G_E_ and G_L_. ICU, intensive care unit. *p* = 0.140 for 90-day survival difference between *G*_E_ and *G*_L_

There were no device-related adverse events reported during the entire study period. Following ICU admission, mortality rates for the overall cohort were 14% at 30 days and 26% at 90 days. The temporal distribution of deaths in the overall cohort and in G_E_ and G_L_ at 30 and 90 days is displayed in the Kaplan–Meier survival curve (Fig. 2). There was no significant difference in survival between G_E_ and G_L_. Individual mortality rates from the 5 participating institutions were each under 50%, ranging from 0% (0/10) to 43% (6/14) without any statistically significant differences between sites (Chi-square test, *p* = 0.51). Finally, following ICU discharge, no deaths occurred and all patients were discharged from the hospital.

## Discussion

There are three major findings in our results. First, adjunct hemoadsorption therapy in hyperinflamed, critically ill COVID-19 patients with ARDS on VV ECMO is easy to implement and safe with no significant device-related adverse events reported for the full study duration. Second, the concurrent use of hemoadsorption with VV ECMO was associated with favorable survival rates. Finally, earlier initiation of VV ECMO with adjunct hemoadsorption may result in faster organ recovery as indicated by significantly shorter duration of mechanical lung support and length of stay in the ICU.

The CTC registry is the only multicenter dataset on the combined use of extracorporeal hemoadsorption with VV ECMO. Previous studies in patients with hyperinflammatory conditions including septic shock, have suggested that early initiation of CytoSorb hemoadsorption may reduce the risk of irreversible organ damage [8-13]. Similarly, in an early report on 26 critically ill COVID-19 patients with shock, severe ARDS requiring VV ECMO, renal failure, and severe hyperinflammation (average IL-6: 1068 ± 1277 pg/mL), the early initiation of CytoSorb hemoadsorption was associated with a significant reduction of inflammatory mediators, marked improvement in lung function, hemodynamic stabilization, and 62.5% survival [12]. Our previously published preliminary results on the first 52 COVID-19 patients enrolled in the CTC registry reported 73% survival at 90 days [6]. The current results from 100 patients confirm our prior observation with 74% survival at 90 days in this extremely high-risk population and also provide some preliminary data suggesting that starting VV ECMO and hemoadsorption early may improve outcomes.

The CTC registry collected data on the real-world performance of extracorporeal hemoadsorption in a well-defined COVID-19 population using a uniform therapeutic approach as defined by the EUA criteria. Detailed information was collected on baseline medical history, comorbidities, concomitant medications, additional therapeutic procedures, CytoSorb use parameters, clinical laboratory parameters and vital status. The information collected in the CTC registry is similar to the data collected by the international ELSO registry that has systematically captured the worldwide experience and outcomes associated with ECMO support in COVID-19 patients [14]. Importantly, the CTC registry population was also very similar to the adult North American cohort of the ELSO COVID-19 registry (November 27, 2020: *n* = 9212) comprising mostly males (CTC: 63%, ELSO: 67%) of fairly young age (CTC: 44 years, ELSO: 47 years) [15]. However, the 26% 90-days mortality reported here is lower than the 48% 90-days mortality reported in the ELSO cohort [15]. Another recent multicenter US study on COVID-19 patients (*n* = 292) requiring ECMO support but without the use of adjunct hemoadsorption, also reported cumulative in-hospital mortality of 42% further corroborating the high risk for death in these critically ill COVID-19 patients [16]. It is noteworthy that the high survival observed in the CTC registry has remained consistent over time and across multiple pandemic waves attributed to different viral strains, which have persisted despite global vaccination efforts and evolving therapeutic approaches. This observation suggests that the combination of severe hypoxemia and hyperinflammation may be a common denominator in the underlying pathophysiology of severe COVID, irrespective of the viral strain.

The CTC results also suggest that using a protocolized approach of early initiation of ECMO and simultaneous initiation of hemoadsorption may be associated with shorter duration of organ support, shorter ICU stay and a trend toward improved survival. However, based on the absence of a comparator group it is not possible to discern whether the favorable outcomes observed are attributable solely to early VV ECMO initiation or early hemoadsorption. Indeed, the results can only be interpreted as attributable to the combination of early initiation of both. Interestingly, *G*_E_ patients had significantly worse baseline respiratory parameters than the *G*_L_ group, which may in part explain the decision for early initiation of hemadsorption. One additional and intriguing consideration is that the earlier treatment initiation in *G*_E_ patients may be a surrogate for a hyperinflamed disease phenotype highlighted by more rapid progression, but also by a better response to cytokine removal. Unfortunately, there is no sufficient information in the current dataset to adequately investigate this hypothesis; however, this hypothesis should be considered for investigation in future research. Nevertheless, the *G*_E_ group had faster weaning from ventilatory and vasopressor support, lower cumulative fluid balance, shorter ICU stays, and higher survival. It is important to note that patients in the *G*_L_ group were also in the ICU and on mechanical ventilation for a significantly longer duration before ECMO initiation compared to those in *G*_E_. These additional factors may each have contributed to the better outcomes seen in the G_E_ group. Previous studies in COVID-19 patients on ECMO also treated with adjunct hemoadsorption have been from single center experiences with relatively small numbers of patients. Not surprisingly, these studies have reported a broad range of outcomes. Rieder et al., reported in a small, randomized sample of 8 patients more pronounced IL-6 removal in the hemoadsorption group, despite higher initial baseline levels [17]. In another recent study by Akil et al. of the 26 patients, 16 patients (58.5 ± 11.7 years old) treated with CytoSorb between March 2020–2021 with severe ARDS who failed mechanical ventilation and proning, with pressor-dependent shock, elevated lactate and IL-6, were immediately placed on VV ECMO on ICU admission and treated with adjunct hemoadsorption (10 integrated with CRRT, 6 with ECMO). Patients had rapid and sustained hemodynamic stabilization, improved control of the hyperinflammatory response and an improvement in oxygenation with a 90-days survival of 62.5% [18]. Meanwhile, Lebreton et al. reported on 11 consecutive prospectively enrolled COVID-19 patients on ECMO and hemoadsorption, and 11 historical controls (overall median age was 49 [33–65]) years old). Although timing of CytoSorb initiation varied widely, 60-day survival in the hemoadsorption group was 72.7% [19].

In somewhat stark contrast, Supady et al. reported that in a single center study involving 17 patients treated with hemoadsorption (median 62.0 [54.0–71.5] years old) versus 17 control patients (59.0 [43.5–66.5] years old), hemoadsorption was associated with worse 30-day survival (18% vs 76%) when used during the first days of ECMO support in COVID-19 [20]. Of note, despite randomization, the small sample size led to significant baseline imbalances between the groups, including greater pressor requirements in the hemoadsorption group as well as markedly higher median D-Dimer baseline levels (9.1 [4.5–21.0] mg/L vs 4.7 [3.4–13.5] mg/L). In previous studies, elevated D-dimer levels have been associated with increased mortality, and suggestive of more advanced COVID-19 disease with thromboembolic complications [21]. More recently, Jarczak et al. [22] reported faster clinical stabilization in patients with CytoSorb in critically ill COVID-19 patients in refractory shock and confirmed hyperinflammation (46% also on ECMO) although these findings were not statistically significant.

Similar to the observations from CTC, a small case series also suggested benefit for early initiation of hemoadsorption therapy in COVID-19 patients who were hemodynamically stable (no vasopressor requirements) and not (yet) intubated [23]. In another case series of 26 consecutive COVID-19 patients with moderate to severe ARDS, overall survival was 80.7%, with the authors noting that the time to hemoadsorption treatment from the onset of symptoms was significantly shorter in the survivor group [24]. This is further supported by another recent case series of 50 critically ill COVID-19 patients [25]. Although only 4 patients received ECMO in this cohort, the overall patient population was very sick with a predicted mortality of around 70%, while actual mortality was 30%. The therapy was commenced within 24 h after a life-threatening state developed, which also supports the benefits of early start of treatment.

It has to be noted that not all the publications discussed above describe concomitant use of CytoSorb and VV ECMO, hence the extent of transferability of the respective results to an ECMO plus hemoadsorption treated population such as in the CTC registry remains unclear. Importantly, also from the discussed reports, it cannot be determined whether it is the early ECMO start alone or the combination of early ECMO with early hemoadsorption that results in the best outcomes.

Of note, the use of adjunct hemoadsorption in these critically ill patients has been described as both easy to implement and safe without any device-related adverse reported to date. Overall, the CTC results support the concept of “enhanced lung rest” that is based on the premise that while ventilation is being handled off-line with ECMO which reduces VILI and allows the lungs to rest, adjunct hemoadsorption could address accompanying systemic hyperinflammation thereby further promoting lung and other organ healing.

### Strengths and limitations

The major limitation of the CTC registry is the lack of a control group that does not permit a comparison regarding efficacy. Furthermore, it is difficult to accurately discern the relative contribution of early ECMO versus early hemoadsorption to the observed overall clinical benefits. An additional limitation is that biomarker data were collected according to standard practice at each institution and are therefore missing at various time points in a large proportion of the study population. Another limitation is that adjustment for potential confounders, such as concomitant medications or specific severity scores, was not possible in the current analysis. Nevertheless, this the largest systematically collected dataset with the combined use of hemoadsorption and ECMO in COVID-19 patients. Additionally, the multicenter design proffers external validity and mitigates the lack of generalizability of single center reports.

## Conclusions

The CTC registry results suggest that adjunct hemoadsorption therapy in combination with VV ECMO in critically ill COVID-19 patients is easy-to-implement, safe, and associated with high survival rates. Early initiation of VV ECMO together with hemoadsorption may further improve outcomes by reducing organ support requirements and length of ICU stay in this extremely sick population. These observations provide some early support for the concept of “enhanced lung rest” with the simultaneous combination of VV ECMO and hemoadsorption, a novel and intriguing therapeutic approach that warrants further investigation in the treatment of severe ARDS.

### Supplementary Information

**Additional file 1**. **Table S1**: IRB statement and site list including approval numbers and approval dates.

## Data Availability

The CTC registry dataset supporting the findings presented in this report is held by CytoSorbents Corporation, the study sponsor. Data are provided, and permission to use was granted after a written request to the sponsor. Requests for registry data should be directed to Dr. Joerg Scheier, Joerg.Scheier@cytosorbents.com.
