# Supplementary material for: Extracorporeal hemoadsorption in critically ill COVID-19 patients on VV ECMO: the CytoSorb therapy in COVID-19 (CTC) registry
Source: Crit Care. 2023 Jun 19;27:243. doi: 10.1186/s13054-023-04517-3 (PMC10280833; doi:10.1186/s13054-023-04517-3)
Supplement: Supplementary file 1 — Additional file 1. Table S1: IRB statement and site list including approval numbers and approval dates. [file 13054_2023_4517_MOESM1_ESM.docx]

**Supplemental Table 1. IRB statement and site list including approval numbers and approval dates**

| Site Number | Site Name | Investigator | IRB Name/ Address | IRB Approval Number | IRB Approval Date |
| --- | --- | --- | --- | --- | --- |
| 01 | New York University | Deane Smith, MD | NYU School of Medicine Science and Research IRB 1 Park Ave, 6^th^ floor New York, NY 10016 | i20-01189 | 13 Aug 2020 |
| 02 | University of Chicago | Tae Song, MD | BSD IRB Committee B Univ. of Chicago Biological Sciences Division/ Univ. of Chicago Medical Center 5841 S. Maryland Ave., MC7132, I-525,  Chicago, IL 60637 | IRB20-1057 | 26 Aug 2020 |
| 03 | Medical College of Wisconsin | Lucian Durham, MD | Medical College of WI/ Froedtert Hospital IRB  9200 W. Wisconsin Ave  Milwaukee, WI 53226 | PRO00038637 | 29 Sept 2020 |
| 04 | West Virginia University | Jeremiah Hayanga, MD | WCGIRB 1019 39^th^ Ave SE/ Ste 120 Puyallup, WA 98374 | 1306178 | 8 Apr 2021 |
| 05 | Franciscan | Andrew Barksdale, MD | WCGIRB 1019 39^th^ Ave SE/ Ste 120 Puyallup, WA 98374 | 1305036 | 29 Jun 2021 |

Site list in order of timing of activation
